# Functional Divergence of Two General Odorant-Binding Proteins to Sex Pheromones and Host Plant Volatiles in *Adoxophyes orana* (Lepidoptera: Tortricidae)

**DOI:** 10.3390/insects16090880

**Published:** 2025-08-24

**Authors:** Shaoqiu Ren, Yuhan Liu, Xiulin Chen, Kun Luo, Jirong Zhao, Guangwei Li, Boliao Li

**Affiliations:** 1 Key Laboratory for Applied Ecology of Loess Plateau (Shaanxi Province), College of Life Sciences, Yan’an University, Yan’an 716000, China; 17710451579@163.com (S.R.);; 2 Shaanxi Key Laboratory of Research and Utilization of Resource Plants on the Loess Plateau, Yan’an University, Yan’an 716000, China

**Keywords:** *Adoxophyes orana*, olfaction, general odorant binding protein, fluresence competitive binding assay, molecular dynamics simulation

## Abstract

*Adoxophyes orana* (Lepidoptera: Tortricidae) is a significant polyphagous leafroller that damages trees and shrubs in Rosaceae and other families. However, the molecular mechanisms by which this pest recognizes sex pheromones and host plant volatiles remain largely unknown. Tissue expression profiles indicated that two general odorant-binding proteins (AoraGOBP1 and AoraGOBP2) were more abundant in the antennae and wings of both sexes, with AoraGOBP1 being rich in the female head and abdomen. Temporal expression profiles showed that AoraGOBP1 was expressed at the highest level in 5 day-nmated adults, while AoraGOBP2 exhibited high expression in 5 day-unmated, 7 day-unmated, and mated female adults. Fluorescence competitive binding assays of heterologous expressed AoraGOBPs demonstrated that AoraGOBP2 strongly bound to the primary sex pheromone Z9-14:Ac, and two minor sex pheromones Z9-14:OH and Z11-14:OH, whereas AoraGOBP1 only showed a high binding affinity to Z9-14:Ac. What is more, AoraGOBP1 exhibited a broader binding spectrum for host plant volatiles than AoraGOBP2. Molecular dockings, molecular dynamic simulations, and per-residue binding free decompositions indicated that the van der Waals interaction was the predominant contributor to the binding free energy. Electrostatic interactions between aldehydes, or alcohols and AoraGOBPs stabilized the conformational structures. Phe12 from AoraGOBP1, and Phe13 from AoraGOBP2 were identified as the most important residues that contributed to bind free energy. Our findings provide a comprehensive insight into the molecular mechanisms of olfactory recognition in *A. orana*, facilitating the development of chemical ecology-based approaches for the control.

## Simple Summary

*Adoxophyes orana* (Lepidoptera: Tortricidae) is a significant leafroller that damages fruit trees in Rosaceae and other plant families. Insects rely on their olfactory system to locate host plants, oviposition sites, and mates. In this study, we assessed tissue and temporal expression profiles of *AoraGOBP1* and *AoraGOBP2*. The binding affinities of recombinant AoraGOBP1 and AoraGOBP2 were determined by fluorescence competition binding assays. Then, key amino acid residues interacting with ligand were identified by molecular docking, molecular dynamics simulations, and per-residue binding free energy decompositions. In general, AoraGOBP2 showed higher binding affinities for sex pheromones, whereas AoraGOBP1 exhibited a wider binding spectrum for host plant volatiles.

## 1. Introduction

Insects rely on their sensitive olfactory system to receive semiochemicals from external environment, to locate host plants, identify mates, and keep away from dangers [1,2]. Numerous studies have demonstrated that chemical odorants, including host plant volatiles (HPVs) and pheromones, served as signals in the behavioral processes [3,4,5]. For example, a mixture of (*Z*)-3-hexenyl acetate, (*Z*)-3-hexenol, (*E*)-2-hexenal, benzaldehyde, and benzonitrile has been reported to attract female *Grapholita molesta* [6,7], while nonanal, a peach-specific aldehyde, also elicits attraction of females of this species [8]. Three isothiocyanates and five green leaf volatiles were found as key olfactory attractants for host location of *P. xylostella* [9]. Therefore, exploring the olfactory system of insects is indispensable for understanding their behavioral patterns.

A set of proteins constitutes the complex olfactory system, involving odorant-binding proteins (OBPs), chemosensory proteins (CSPs), olfactory receptors (ORs), gustatory receptors (GRs), ionotropic receptors (IRs), and sensory neuron membrane proteins (SNMPs) [10]. When hydrophobic olfactory molecules enter the antenna lymph, they were wrapped and transported by OBPs or CSPs, which subsequently deliver them to ORs and GRs in sensory neurons [1,10]. OBPs in Lepidoptera have been generally classified into pheromone binding proteins (PBPs), general odorant-binding proteins (GOBPs), and antennal-binding proteins (ABPs). PBPs participate in sex pheromone recognition, while GOBPs are traditionally associated with host plant volatile detection [11]. However, in addition to binding host plant volatiles, an increasing number of studies have revealed that GOBPs are also involved in recognizing sex pheromones and insecticides [12,13,14,15,16]. For example, GOBP1 played a key role in semiochemical recognition of *Conogethes punctiferalis* [12]. AlepGOBP2 in *Athetis lepigone* showed high binding affinities to two pheromone components, two maize plant volatiles and two insecticides, while AlepGOBP1 bound ocimene and two insecticides [13]. All three GfunGOBPs in *G. funebrana* strongly bound sex pheromones, and GfunGOBP1 and GfunGOBP3 also exhibited affinity for insecticides [14].

The summer fruit tortrix moth, *Adoxophyes orana* (Lepidotera: Tortricidae), distributed in Asia and Europe, is a polyphagous leafroller species that damages trees and shrubs in Rosaceae and other families [17,18]. Although trees and shrubs can tolerate limited larvae feeding and leaf rolling, high larvae densities lead to loss of fruit production by larvae chewing. It is imperative to develop eco-friendly approaches to control *A. orana*, including traps with sex pheromone causing mate-disruption in orchards [19,20,21]. Previous researchers have determined the sex pheromone of *A. arona*, consisting of (*Z*)-9-tetradecenyl acetate (Z9-14:Ac), (*Z*)-11-tetradecenyl acetate (Z11-14:Ac), (*Z*)-9-tetradecen-1-ol (*Z*9-14:OH), and (*Z*)-9-tetradecen-1-ol (*Z*11-14:OH), with the former two serving as primary components and the latter two as secondary components [22,23,24]. However, limited understanding of olfactory mechanism of *A. orana* and other leafroller species restricts the development of control Tortricinae species. As far as we know, it seems that there are no reports on the molecular mechanisms of olfactory recognition in Tortricinae species based on the binding affinity of recombinant odorant-binding proteins. Relatively few studies involved in the molecular dynamic simulation compare the homologous modeling and molecular docking when exploring the interactions between proteins and ligands [16]. Herein, we comprehensively determined the expression of GOBPs across different developmental stages and in various adult tissues. Then, we characterized the binding affinities of AoraGOBP1 and AoraGOBP2 to sex pheromones and HPVs. Molecular docking, combined with molecular dynamics simulations, further revealed critical amino acid residues involved in GOBP–ligand interaction, providing new insights into the molecular basis of olfaction in Tortricinae pest species.

## 2. Materials and Methods

### 2.1. Insect Rearing

The larvae of *Adoxophyes orana* tested were originally collected from apple orchards in Ganquan, Shaanxi, China (109°22′10″ E, 36°22′34″ N) in July in 2018 [19]. Larvae were fed on fresh mulberry leaves in an 800 mL polyethylene cuboid box (16 cm × 10 cm × 5 cm). Pupae were collected into polyethylene cups. The emerged adults were fed on 5% sugar solution. The insects were reared in an artificial climate chamber with the following conditions: (24 ± 1) °C, (60 ± 5)%, and L:D = 15 h:9 h. Approximately two days after emergence, the adults could mate.

### 2.2. RNA Extraction, cDNA Synthesis, and qRT-PCR

Total RNA for *GOBPs* amplification were extracted from antennae from about approximately 100 pairs of adults 3 days after eclosion following the protocols of AG RNAex reagent (Accurate Biology, Changsha, China). For tissue expression profile, total RNA was extracted from about 100 pairs of antennae, heads or thorax of 10, abdomen from 5, legs from 25, and wings from 20 adults. For temporal expression profile, total RNA was expected from eighty eggs, sixty 1st-instar larvae, thirty 2nd-instar larvae, fifteen 3rd-instar larvae, ten 4th-instar larvae, five 5th-instar larvae, or four pupae on the 2nd and 6th day after pupation. First-strand cDNA was generated following the instructions of HiScript III 1st Strand cDNA Synthesis Kit (+gDNA wiper) (Vazyme, Nanjing, China). The RT-qPCR analyses were conducted on StepOnePlus™ Real-Time PCR System (ABI, Carlsbad, CA, USA) using 2 × Q3 SYBR qPCR Master Mix (TOLOBIO, Shanghai, China) based on the manufacturer’s protocols. Each Rt-qPCR reaction was conducted in a total volume of 20 µL mixture containing 10 µL 2 × Q3 SYBR qPCR Master Mix, 0.4 µL of each primer (10 µM), 1 µL of diluted cDNA, and 8.2 µL of sterile water. The amplification procedure was as follows: 95 °C for 30 s, followed by 40 cycles at 95 °C for 10 s, and 60 °C for 30 s. All developmental stages and tissues were performed with three biological replications and three technical duplicates. *β-actin* and *EF1-α* were used as reference genes. The relative expressions of *GOBPs* were calculated using the 2^−ΔΔCt^ method [25], and normalized using the geometric means of the two reference genes.

### 2.3. Signal Peptides Prediction, Sequence Alignment and Phylogenetic Analyses

The signal peptides of AoraGOBPs and GOBPs from other moths were predicted using SignalP 6.0 [26]. These sequences after the signal peptides have been removed were aligned using MUSCLE (v. 3.8.1551) [27] and further edited with the WebLogo Online program (https://weblogo.berkeley.edu/logo.cgi) (accessed on 15 February 2025) [28]. Phylogenetic analyses were performed using iqtree2 (v. 2.0.7) [29] and were then visualized with iTOL v6 [30].

### 2.4. Prokaryotic Expression and Purification of rAoraGOBPs

Specific primers incorporating restriction enzyme sites were designed to amplify the DNA fragments encoding AoraGOBPs without signal peptides. PCR-amplified AoraGOBPs were verified by 1.0% agarose gel and extracted using a Gel Extraction Kit (BioFlux, Hangzhou, China). The purified products were ligated into the 5×TA/Blunt-Zero Cloning Kit (Vazyme, Nanjing, China), and transformed into DH5α competent cells (TOLOBIO, Beijing, China). Positive clones were then screened by PCR. Plasmids containing the AoraGOBPs (pMD™19-T-AoraGOBPs) were extracted using the FastPure Plasmid Mini Kit (Vazyme, Nanjing, China). After verification, pMD™19-T-AoraGOBPs plasmids were subjected to double-digestion with restriction endonucleases HandIII and BamHI for 4 h at 37 °C. Recombinant AoraGOBPs fragments were ligated into the expression vector pET28a (+) using T4 DNA ligase (ThermoFisher Scientific, Vilnius, Lithuania) at 4 °C overnight.

The pET28a(+)-GmolGOBPs plasmids were extracted and transferred into *BL21* competent cells (TransGen Biotech., Beijing, China). The cells were cultured in LB liquid medium with kanamycins (50 μg/mL) for 15 h under oscillatory conditions. When the culture OD_600_ reached 0.6 to 0.8, the cells were induced to expression by adding isopropyl-β-D-thiogalactoside (IPTG) overnight at 28 °C and 220 rpm to a final concentration of 0.5 mM. The rAoraGOBPs were validated using SDS-PAGE and identified as inclusion bodies. The bacterial cells were harvested via centrifugation at 4 °C (8000 rpm, 5 min). After proteins were purified by a standard Ni-NTA agarose magnetic beads (7sea biotech, Shanghai, China) to obtain a soluble protein of about 17 kDa in size, the proteins were recovered in 20 mM Tris-HCl (pH 7.4) by dialysis and used for in vitro fluorescence competitive binding assays. The purity and concentration of rAoraGOBPs was verified by SDS-PAGE and BCA protein assay kit (Vazyme, Nanjing, China), respectively.

### 2.5. Fluorescence Competitive Binding Assay

The fluorescent competitive binding assays were conducted using an F-2700 spectrophotofluorometer (Hitachi, Tokyo, Japan) to evaluate the binding affinities of AoraGOBP1 and AoraGOBP2 to sex pheromones, and HPVs. At the beginning, the fluorescence probe 1 − NPN and the tested ligands were dissolved in chromatographic methanol to create 100 mM stock solution, which were subsequently diluted to 1 mM for assays. rAoraGOBP were diluted to 2 μM in 20 mM Tris-HCl buffer (pH 7.4), and 2 mL of this solution was titrated with 1 − NPN to a final concentration of 18 μM, and the binding affinity was determined by measuring fluorescence intensity (excitation at 337 nm, emission scanned from 370 to 550 nm). With the increase of 1 − NPN concentration, the change in fluorescence intensity decreased and eventually stabilized, and the data were recorded. Binding dissociation constant *K*_1−*NPN*_ (*K_d_*) of 1 − NPN to rAoraGOBPs were determined. Then, 2 mL protein solution containing 2 μM rAoraGOBPs was titrated with 1 μM of each ligand to a final concentration of 18 µM. The fluorescence intensity was measured and recorded after a 2 min reaction. The *K_i_* for competitive binding of each ligand to rAoraGOBPs using the formula *K_i_* = [*IC*_50_]/(1 + [1 − NPN]/*K*_1−*NPN*_), where *IC*_50_ is the ligand concentration corresponding to 50% displacement of 1 − NPN. Each assay was replicated three times biologically. The *K_d_* and *K_i_* are presented as *mean* ± *S.E.M*.

### 2.6. Homology Modeling, Molecular Docking, and Molecular Dynamic Simulation

The 3D structures of AoraGOBP1 and AoraGOBP2 were constructed on ColabFold server (MIT, Cambridge, USA) [31,32]. The 3D structures of the ligands were downloaded from PubChem [33]. Molecular docking was applied on the CB-Dock 2 server with the default settings [34]. The interactions of AoraGOBP–ligand complexes were visualized by PyMOL-open-source v2.5.0 [35] and Ligplot+ (v. 2.2.8) [36].

The MD simulations of AoraGOBP–ligand complexes were carried out for 200 nanoseconds (ns) using GROMACS (v. 2024.4) [37] with the AMBER99SB protein forcefield [38]. The simulation started with solvating the complex to neutralize the system. Then, energy minimization was achieved using the steepest descent algorithm with a step size of 0.01 nm. System equilibration was performed under *NVT* and *NPT* ensembles. After that, a 200 ns production molecular dynamic was performed. Analyses of root-mean-square deviation (RMSD) and root-mean-square fluctuation (RMSF) were conducted using GROMACS (v. 2024.4) built-in tools [37], and visualized using *ggplot2* package (v. 3.4.3) [39] in R (v. 4.3.2). Finally, the binding energies for protein and ligands were calculated using the gmx-MMPBSA tool (v. 1.5.7) [40].

### 2.7. Statistic Analysis

Statistical analyses for the results of RT-qPCR and fluorescence competitive binding assays were conducted using Graphpad Prism 6.0. Every data point is presented as the *mean* ± *S.E.M* with three replicates. Normality in each group and homogeneity of variance were tested before perimetric analyses. One-way ANOVA with Tukey’s HSD comparison was employed to assess differences among multiple samples, with significance set at *p* < 0.05. Student’s *t*-test was used to compare differences between two sexes (**: *p* < 0.01; *: *p* < 0.05; ns: *p* ≥ 0.05).

## 3. Results

### 3.1. Sequence Analysis of AoraGOBP1 and AoraGOBP2

**Figure 1:**
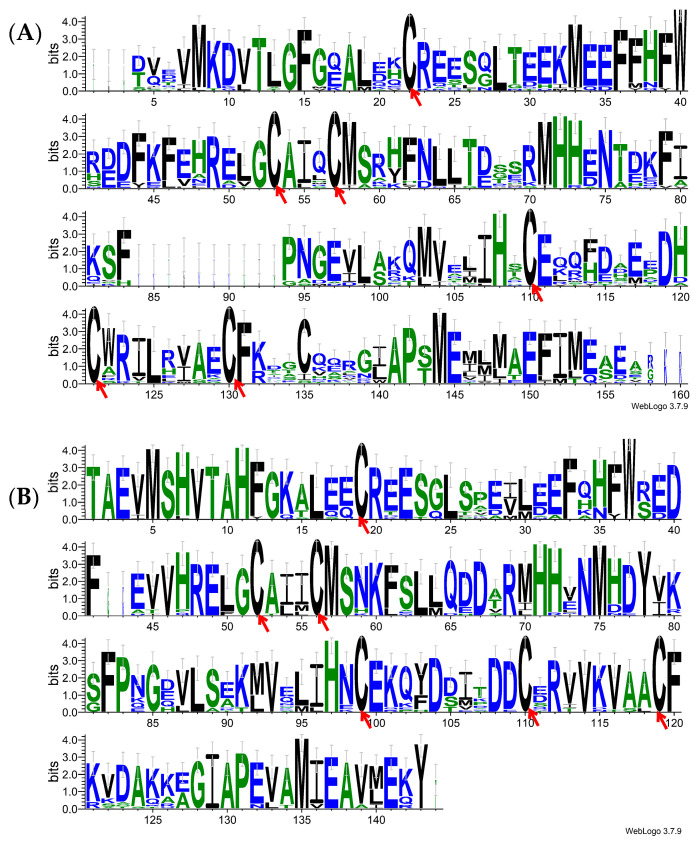
Alignment of AoraGOBPs and GOBPs from the other 19 lepidopteron species. (**A**) GOBP1; (**B**) GOBP2. The red arrows at the bottom indicate conserved cysteines. The insect species and GeneBank accession numbers and signal peptide sequences are listed in Table S2.

**Figure 2:**
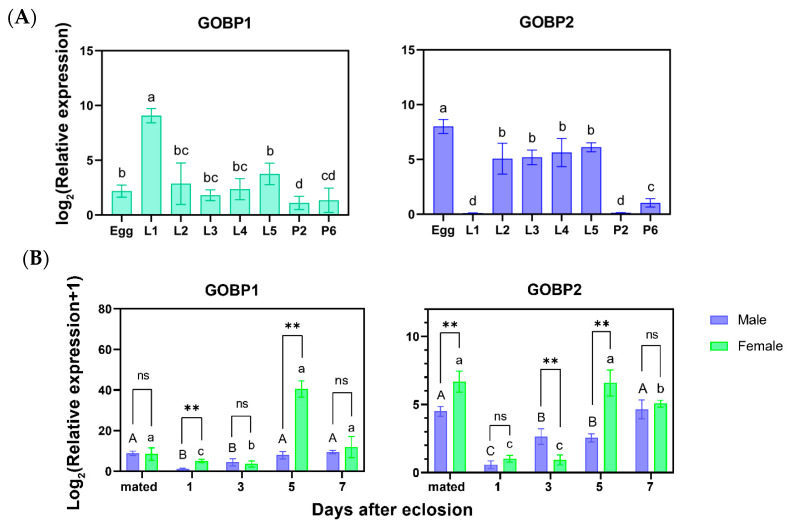

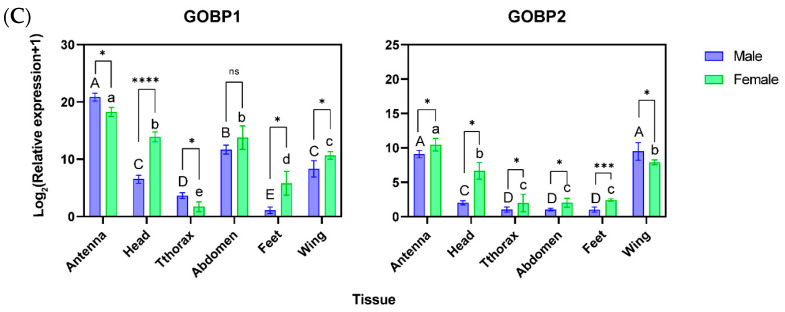
Relative expression of *AoraGOBP1* and *AoraGOBP2* from *A. orana* using qRT-PCR. (**A**) Various immature developmental stages. (**B**) Different ages and mating status of female and male adults. (**C**) Different tissues of female and male adults. Different upper- and lower-case letters indicate significant differences among different samples using Tukey’s multiple comparisons after one-way ANOVA (*p* < 0.05). *ns*, *, **, ***, and **** mean there were significant differences (*p* ≥ 0.05, *p* < 0.05, *p* < 0.01, *p* < 0.001, and *p* < 0.0001, respectively) between sexes in the same tissue, or between sexes on the same day after eclosion. Error bar represents *S.E.M*.

The ORF of *AoraGOBP1* and *AoraGOBP2* cover 492 and 486 bp, encoding 163 and 161 amino acid residues, respectively. The signal peptides of AoraGOBP1 and AoraGOBP2 were composed of 19 and 20 amino acids, respectively (Figure S1). The mature AoraGOBP1 has a predicted molecular weight (*MW*) of 16.82 kDa and an isoelectric point (*pI*) of 5.10. The mature AoraGOBP2 has a *MW* of 16.17 kDa and a *pI* of 5.10. Both AoraGOBPs have six conserved cysteines (Figure 1). The 3D structures of AoraGOBP1 and AoraGOBP2 contain seven α-helices: Val2-Ser23 (α1), Glu27-His35 (α2), Arg46-Phe59 (α3), His70-Ser79 (α4), Gly83-His101 (α5), His107-Arg125 (α6), and Met101-Glu143 (α7) for AoraGOBP1 (Figure 2A), and Ala2-Ser23 (α1), Pro27-His35 (α2), Arg46-Phe59 (α3), His70-Ser79 (α4), Gly83-Tyr100 (α5), Asp107-Ala125 (α6), and Val131-Lys140 (α7) for AoraGOBP2 (Figure 2B). Phylogenic analysis indicated GOBP1 and GOBP2 were distributed in different branches (Figure S2).

### 3.2. Spatial–Temporal Patterns of OBPs in A. orona

For immature stages, expression of *AoraGOBP1* was significantly higher in first-instar larvae than in the other developmental stages, while *AoraGOBP2* was abundantly expressed in eggs, and poorly expressed in first-instar larvae and 2-day-old pupae (Figure 2A). The expression of *AoraGOBP1* in adults was significantly elevated in 5 day- and 7 day-unmated males after eclosion compared to 1 day-unmated, 3 day-unmated, and mated males, while its expression of females kept relatively stable. The expression of *AoraGOBP2* was higher on 5 day-unmated, 7 day-unmated, and mated adults for both sexes than in 1 day-unmated and 3 day-unmated adults (Figure 2B). Regarding tissue expression patterns in adults, both *AoraGOBPs* exhibited maximal expression in antennae, although *AoraGOBP2* was also abundantly expressed in wings. What is more, *AoraGOBP1* showed rich expression in female heads and abdomens in both sexes (Figure 2C).

### 3.3. Expression and Purification of rAoraGOBPs

**Figure 3:**
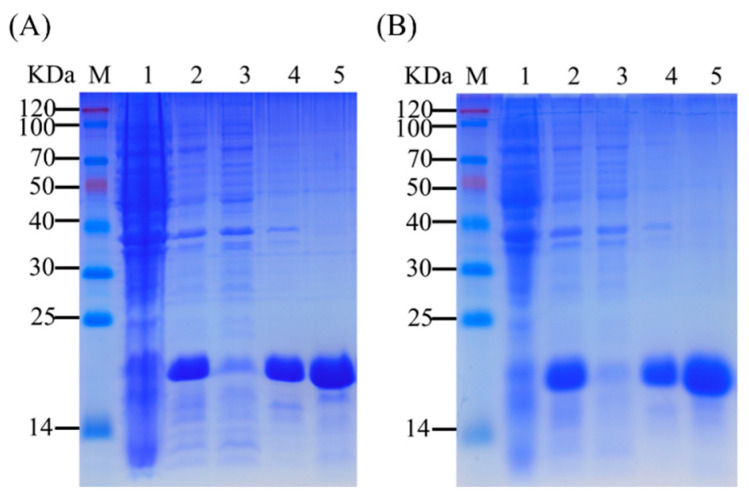
SDS-PAGE analysis of rAoraGOBPs. (**A**) rAoraGOBP1; (**B**) rAoraGOBP2. Lane M, protein molecular weight marker; Lane 1 and 2, before and after IPTG-induced pET28a(+)-AoraGOBP1/2; Lane 3, supernatant of IPTG-induced pET28a(+)-AoraGOBP1/2; Lane 4, inclusion bodies of IPTG-induced pET28a(+)-AoraGOBP1/2. Lane 5, purified rAoraGOBP1/2.

rAoraGOBP1 and rAoraGOBP2 were successfully expressed in *BL21* after IPTG induction. These two proteins were mainly expressed in inclusion bodies (Figure 3). SDS-PAGE results showed that each of the purified rAoraGOBPs migrated as a single band with an MW of approximately 17 kDa (Figure 3).

### 3.4. Ligand Binding of rAoraGOBPs

Fluorescent binding assays showed that *K_d_* for rAoraGOBP1 of 18.36 μM (95% CI: 15.59~21.86 μM), and 1.65 μM (95% CI: 1.49~1.88 μM) for rAoraGOBP2, suggesting 1 − NPN as a suitable fluorescent probe. Then, we accessed the binding affinities of both rAoraGOBPs towards 55 chemical ligands, including five sex pheromone components and 51 HPVs. rAoraGOBP2 showed strong binding affinity for *Z*9-14:OH (*K_i_* = 1.49 ± 0.01 μM) and *Z*11-14:OH (*K_i_* = 1.21 ± 0.02 μM), along with moderate binding affinity to *Z*9-14:Ac, whereas rAoraGOBP1 showed no binding affinity to sex pheromone (Table 1, Figure S3A). Regarding host plant volatiles, rAoraGOBP1 displayed strong binding affinities to decanal (*K_i_* = 0.78 ± 0.15 μM) and methyl salicylate (*K_i_* = 4.41 ± 0.20 μM), moderate binding affinities to pear ester (*K_i_* = 5.08 ± 0.13 μM) and *Z*-3-hexen-1-yl 3-methylbutanoate (*K_i_* = 6.21 ± 0.18 μM), and weak binding affinities to five other volatile host plants. rAoraGOBP2 strongly bound farnesol (*K_i_* = 1.13 ± 0.02 μM) and 12:OH (*K_i_* = 1.74 ± 0.03 μM), with moderate affinity to 1,2-Benzenedicarboxylic acid dibutyl ester (*K_i_* = 5.33 ± 0.04 μM) and farnesene (*K_i_* = 6.96 ± 0.32 μM) (Table 1, Figure S3B).

### 3.5. Protein Structure Modeling and Molecular Docking

**Figure 4:**
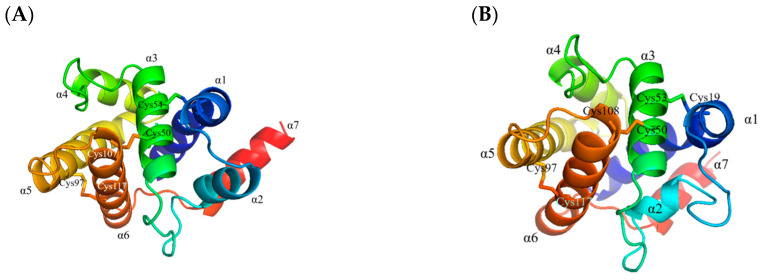
The 3D structure of (**A**) AoraGOBP1, and (**B**) AoraGOBP2 by alphafold2 modeling.

The homologous 3D model of AoraGOBP1 and AoraGOBP2 showed that both AoraGOBPs shared seven α-helicals (α1–α7) (Figure 4). Ramachandran plots verified the stereochemical validity of both protein models (Figure S4). Three disulfide bridges in each AoraGOBP stabilized the 3D structures (Figure 4).

**Figure 5:**
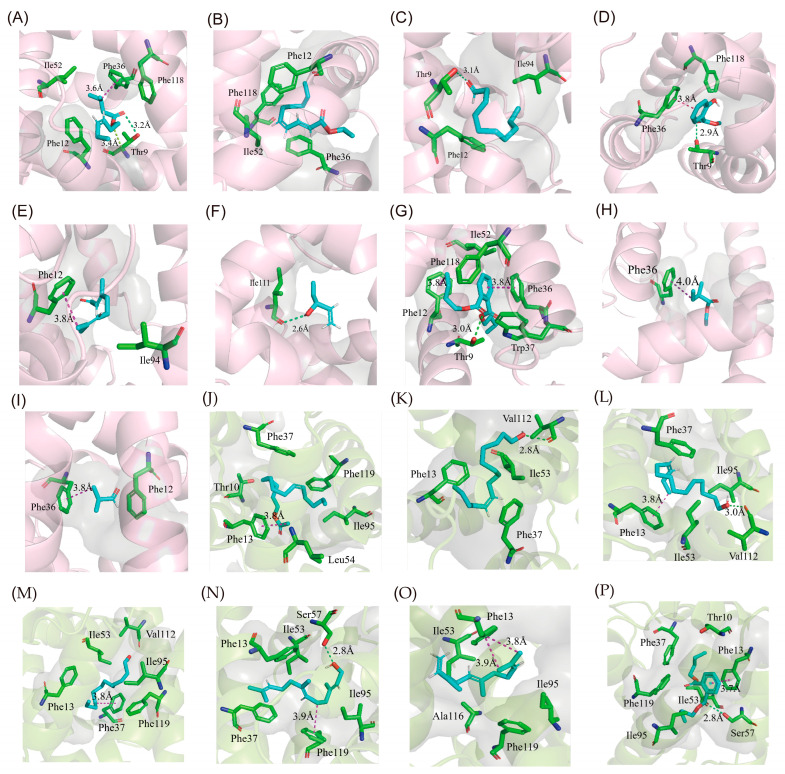
Interactions of the AoraGOBP–ligand complexes. (**A**) AoraGOBP1–cis-3-Hexenyl isovalerate. (**B**) AoraGOBP1–Pear ester. (**C**) AoraGOBP1–Decanal. (**D**) AoraGOBP1–Methyl salicylate. (**E**) AoraGOBP1–Citral. (**F**) AoraGOBP1–1-penten-3-ol. (**G**) AoraGOBP1–Dibutyl Phthalate. (**H**) AoraGOBP1–Ethyl 2-methylbutyrate. (**I**) AoraGOBP1–Isobutyraldehyde. (**J**) AoraGOBP2–Z9-14:Ac. (**K**) AoraGOBP2–Z9-14:OH. (**L**) AoraGOBP2–Z11-14:OH. (**M**) AoraGOBP2–12:OH. (**N**) AoraGOBP2–Farnesol. (**O**) AoraGOBP2–Farnesene. (**P**) AoraGOBP2–Dibutyl Phthalate. The green, magenta, and purple dashed lines represent hydrogen bonds, π-π stacks, and π-σ interactions, respectively.

Based on the results of fluorescence competitive binding assays, we used molecular docking to investigate the interactions of GOBP–ligand complexes. The docking analyses indicated that several nonpolar amino acid residues formed a hydrophobic cavity, ensuring the binding of GOBP to hydrophobic ligand molecules (Figure 5 and Figure S5). In addition, specific residues (such as Thr, Ser, and Val) in the complexes formed hydrogen bonds with ligands; Thr9 in AoraGOBP1 interacted with decanal, methyl salicylate, and dibutyl phthalate. In AoraGOBP2, Val112 formed hydrogen bonds with Z9-14:OH and Z11-14:OH, respectively, and Ser 57 with farnesol and dibutyl phthalate.

### 3.6. Per-Residue Free Energy Decomposition

**Figure 6:**
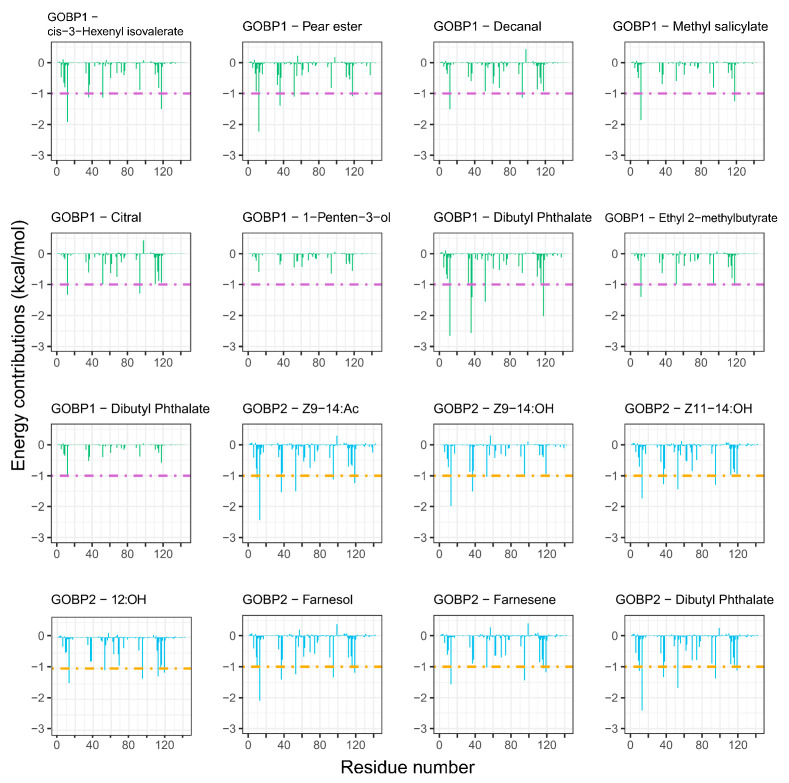
The per-residue free energy decomposition of AoraGOBP1–ligand complexes (green bar) and AoraGOBP2–complexes (blue bar). Dashed lines indicate total binding energy below -1.00 kcal/mol.

Per-residue free energy decomposition analyses (Table 2) showed that Phe12, Phe36, Ile52, Ile94, and Phe118 contributed the most to the binding free energy in the bindings of AoraGOBP1 to different ligands (Figure 6. Similarly, Phe13, Phe37, Ile53, Val112, and Phe119 played a critical role in the binding of AoraGOBP2 to sex pheromones and HPV ligands (Figure 6. These hydrophobic amino acid residues exhibited relatively low RMSF values (Figure S7), confirming the stability of GOBP–ligand interactions.

## 4. Discussion

The tissue expression patterns *AoraGOBPs* suggested that *AoraGOBP1* and *AoraGOBP2* were richly expressed in antennae and moderately expressed in wings. In addition, *AoraGOBP1* was also expressed in the head and abdomen. *GOBP* genes were specifically expressed in several lepidopteran species, such as *Spodoptera frugiperda* [15], *Orthaga achatina* [41], and *Glyphodes pyloalis* [42]. *EoblGOBP2* was highly expressed in the female abdomen, legs, and wings of *Ectropis obliqua*, except the antennae [43]. Temporal expression profiles showed that *AoraGOBP1* was expressed in first-instar larvae, and *AoraGOBP2* in the eggs of second- to fifth-instar larvae. Expression of *SfruGOBPs* also showed expression difference in *S. frugiperda*, where *SfruGOBP1* was detected after pupation, and *SfruGOBP2* was detected after egg hatching [44]. *SlitGOBP2* was highly expressed in first-instar larvae and adults of *S. litura* [45].

OBPs were traditionally classified into three subfamilies: PBPs, GOBPs, and ABPs. PBPs were well-known for their abilities to bind sex pheromones, and GOBPs were usually considered to bind host plant volatiles. However, with the wide application of genomic sequences, it has been observed that GOBPs and PBPs in Lepidoptera were closely located in the same chromosome [15,46], suggesting the role of GOBPs in recognizing sex pheromones. Fluorescent competing binding assays showed that AoraGOBP1 and AoraGOBP2 exhibited functional differentiation. AoraGOBP2 strongly bound two secondary sex pheromone components, *Z*9-14:OH and *Z*11-14:OH, and weakly bound Z9-14:Ac, while AoraGOBP1 did not. AoraGOBP1 exhibited a broader binding spectrum for host plant volatiles than AoraGOBP2, including one alcohol, three aldehyde, and five esters (Table 1). The functional divergence of GOBPs has been observed in other lepidopteran species. For instance, AlepGOBP2 in *A. lepigone* showed high binding affinity to *Z*7-12:Ac and *Z*9-14:Ac, while AlepGOBP1 did not bind to the sex pheromone [13]. Similarly, SfruGOBP2 in *S. frugiperda* exhibited strong binding affinities to *Z*7-12:Ac, *Z*9-12:Ac, *Z*9-14:Ac and *Z*9,E12-14:Ac, while SfruGOBP1 did not bind to the sex pheromone [15]. AipsGOBP2 in *A. ipsilon* had strong binding affinities to *Z*7-12:Ac and *Z*9-14:Ac, whereas AipsGOBP1 showed moderate binding to five sex pheromones [47]. SexiGOBP2 showed strong binding to five sex pheromone components, and SexiGOBP1 only showed weak binding to alcohol sex pheromone compounds [48]. GmolGOBP1 in *G. molesta* exhibited broad binding affinities for both HPVs and primary sex pheromone, while GmolGOBP2 demonstrated specific binding affinity for 12:OH, the minor sex pheromone, and a narrow spectrum of HPVs [49]. It is interesting that AoraGOBP2 had a high affinity to 12:OH. We speculated that maybe *A. orana* relies on AoraGOBP2 to recognize signals from *G. molesta* in the same orchard to avoid niche competition. This speculation needs to be tested using behavior experiments both in the laboratory and in the field. Taken together, the primary function of AoraGOBP1 was the binding of host plant volatiles, whereas AoraGOBP2 was more involved in recognizing sex pheromones.

MD simulations combined with per-residue energy decompositions revealed that the binding energy of *Z*9-14:Ac interacting with the following residues surpassed −1.00 kcal/mol: Phe12, Phe36, Trp37, Ile52, and Phe118 in AoraGOBP1, and Thr10, Phe13, Phe37, Leu54, Ile95, and Phe119 in AoraGOBP2 (Figure 6). In addition, *Z*9-14:Ac interacted with the benzene ring of Phe36 and Phe118 in AoraGOBP1, and of Phe13 from AoraGOBP2 by π-σ interactions (Figure 5A,I). Site-directed mutagenesis of SfruGOBP2 from *S. frugiperda* demonstrated that mutations of Thr9, Phe12, Phe33, Phe36, or Phe118 reduced or even abolished their ability to bind *Z*9-12:Ac, *Z*7-14:Ac, *Z*9-14:Ac, and *Z*9,*E*12-14:Ac [15]. AoraGOBP2 showed strong binding affinities to *Z*9-14:OH and *Z*11-14:OH. Although the total binding energies of AoraGOBP2–Z9-14:OH and AoraGOBP2–Z11-14:OH were higher than those of AoraGOBP2–*Z*9-14:Ac, hydrogen bonds were formed between the oxygen atom in the sidechain of Val112 and each of the hydroxyl group of *Z*9–14:OH and Z11–14:OH (Figure 5K,L). The binding energies of Phe13, Phe37, and Ile53 in these two complexes were below −1.00 kcal/mol. In *M. separata*, the sex pheromone *Z*11-16:Ald was stabilized by seven hydrophobic residues, each contributing a free energy below −0.95 kcal/mol, while a hydrogen bond was formed between Ser56 and *Z*11-16:Ald [16]. Therefore, similar patterns were observed in lepidopteran GOBPs that highly hydrophobic residues, especially Phe, created a binding pocket to stabilize the ligands. When sex pheromone molecules contain hydroxyl or aldehyde carbonyl groups, hydrogen bonds enhance the stability of the protein–ligand complex.

Interestingly, AoraGOBP2 strongly bound *Z*9-14:OH and *Z*11-14:OH, while AoraGOBP1 did not. This could be contributed to the differences in the 3D structures. Distance between each of the 9 amino acid residues from AoraGOBP1 and Z9-14:OH (Met5, Thr9, Phe12, Phe36, Trp37, Ile52, Phe76, Ala115, and Phe118) were within 4 Å, whereas distance between the 11 amino acid residues from AoraGOBP2 and *Z*9-14:OH (Thr10, Phe13, Phe34, Phe36, Trp37, Ile53, Ile95, Val112, Val115, Ala116, and Phe119) were within 4 Å. As for residues within 4 Å away from *Z*11-14:OH, 10 residues (Val8, Thr9, Phe12, Gly13, Phe33, Phe36, Trp37, Ile52, Ala115, Phe118) were found in AoraGOBP1 and 11 in AoraGOBP2 (Thr10, Phe13, Phe34, Phe36, Trp37, Ile53, Met 91, Val112, Val115, Ala118, Phe119). In addition, α7 of AoraGOBP1 seems far away from the other six helices, while this phenomenon does not exist in AoraGOBP2 (Figure 4). Most of the residues above belong to hydrophobic amino acids, which formed hydrophobic pockets. Therefore, hydrophobic pocket in AoraGOBP2 could tightly wrap the minor sex pheromone components more than the pocket in AoraGOBP1.

AoraGOBP2 showed strong binding affinities to farnesene and farnesol (Table 1), two terpenoid biosynthesized from mevalonate. Previous studies have demonstrated that farnesene stimulated oviposition of *Ostrinia nubilalis*, while farnesol preceded the oviposition [50]. Larvae of *Plutella xylostella* were strongly attracted by farnesol [51]. α-farnesene, a compound from apple skin, has been shown to be an attractant of larvae of *Cydia pomonella*. Female adults of *C. pomonella* were attracted to low concentrations of α-farnesene and repelled by high concentrations [52], whereas males showed no significant behavioral response over a wide range of doses [53]. Molecular dockings, MD simulations, and per-residue free energy decompositions revealed that Phe13, Ile53, Ile95, Ala116, and Phe119 in AoraGOBP2 strongly interacted with farnesene via van der Waals forces (Figure 5 and Figure 6). A similar interaction pattern was observed for farnesol, with the highest energy contributions coming from Phe13, Phe37, Ile53, Ile95, and Phe119 in AoraGOBP2 (Figure 6). Ser57 from AoraGOBP2 formed strong hydrogen bonds with farnesol, with a bond distance of 2.8 Å, respectively (Figure 5). Therefore, hydrophobic interactions primarily stabilize the AoraGOBP2–farnesol complex, while hydrogen bonds enhance the conformational stabilities.

## 5. Conclusions

AoraGOBP1 displayed high binding affinities for multiple host plant volatiles, including Decanal, Methyl salicylate, Pear ester, and (*Z*)-3-hexen-1-yl 3-methylbutanoate. In contrast, AoraGOBP2 strongly bound to sex pheromone components, *Z*9-14:OH, *Z*11-14OH, and *Z*9-14:Ac, as well as some host plant components, including farnesol, farnesene, and Dibutyl phthalate. van der Waals forces between hydrophobic residues and ligands contributed the majority of binding free energy in these protein–ligand complexes. Although for the limitations of this study, as a lack of behavior assays for insects whose *AoraGOBP* genes were interfered with dsRNA, these findings provide a comprehensive understanding of the molecular mechanisms underlying olfactory recognition in *A. arona*.

## Figures and Tables

**Table 1:** Binding affinities of AoraGOBPs to sex pheromones and host plant volatiles.

Ligand Name	CAS Number	*K_i_* (μM)		Ligand Name	CAS Number	*K_i_* (μM)	
		GOBP1	GOBP2			GOBP1	GOBP2
*Z*9-14:Ac	35153-15-2		6.46 ± 0.18	1-hexanol	111-27-3	-	-
*Z*11-14:Ac	20711-10-8	-	-	Benzyl alcohol	100-51-6	-	-
*Z*9-14:OH	35153-15-2	-	1.49 ± 0.01	(*Z*)-3-hexen-1-ol	928-96-1	-	-
*Z*11-14:OH	34010-15-6	-	1.24 ± 0.02	Farnesol	4602-84-0		1.13 ± 0.02
*E*9-14:Ac		-	-	Nerolidol	3790-78-1	-	-
12:OH	88170-32-5	-	1.74 ± 0.03	1-Penten-3-ol	616-25-1	10.19 ± 0.14	
Butyl butanoate	109-21-7	-	-	Linalool	78-70-6	-	-
Methyl salicylate	119-36-8	4.41 ± 0.20	-	Heptan-1-ol	111-70-6	-	-
ethyl acetate	141-78-6	-	-	Decan-1-ol	112-30-1	-	-
Ethyl valerate	539-82-2	-	-	Cineole	470-82-6	-	-
Dibutyl phthalate	84-74-2	10.27 ± 0.07	5.33 ± 0.04	(*Z*,*E*)-phytol	7541-49-3	-	-
Ethyl isovalerate	108-64-5	-	-	Isooctyl alcohol	26952-21-6	-	-
cis-3-Hexenyl butyrate	16491-36-4	-	-	Farnesene	502-61-4		6.96 ± 0.32
Methyl oleate	112-62-9	-	-	Camphene	5794-04-7	-	-
Butyl acetate	123-86-4	-	-	(Z)-β-ocimene	13877-91-3	-	-
Ethyl Butyrate	105-54-4	-	-	α-Pinene	7785-70-8	-	-
Pear ester	3025-30-7	5.08 ± 0.13	-	β-Caryophyllene	87-44-5	-	-
Ethyl Tiglate	5837-78-5	-	-	3-carene	4497-92-1	-	-
Isoamyl acetate	123-92-2	-	-	2-methylprop-1-enylbenzene	768-49-0	-	-
Methyl palmitate	112-39-0	-	-	α-phellandrene	99-83-2	-	-
Butyl propanoate	590-01-2	-	-	3,7-Dimethyl-2,6-octadienenitrile	5146-66-7	-	-
(Z)-3-Hexen-1-yl 3-methylbutanoate	35154-45-1	6.21 ± 0.18	-	Benzonitrile	100-47-0	-	-
cis-3-hexenyl acetate	1708-82-3	-	-				
Ethyl hexanoate	123-66-0	-	-				
Ethyl 2-methylbutanoate	7452-79-1	11.78 ± 0.08	-				
Heptanal	111-71-7	-	-				
Hexanal	66-25-1	-	-				
Decanal	112-31-2	0.78 ± 0.15	-				
Benzaldehyde	100-52-7	-	-				
Trans-2-Hexenal	6728-26-3	-	-				
Citral	5392-40-5	11.70 ± 0.44	-				
Nonanal	124-19-6	-	-				
Isobutyraldehyde	78-84-2	11.88 ± 0.41	-				

**Table 2:** Binding free energy for the GOBP–ligand complexes (kcal/mol).

Complex	Electrostatic Energy (Δ*G_ele_*)	van der Waals Energy (Δ*G_vdw_*)	Polar Solvation Energy (Δ*G_PB_*)	Nonpolar Solvation Energy (Δ*G_SA_*)	Total Binding Energy (Δ*G_bind_*)
AoraGOBP1–*cis*-3-Hexenyl isovalerate	−1.89 ± 0.01	−31.21 ± 0.01	8.96 ± 0.01	−4.49 ± 0.00	−28.62 ± 0.02
AoraGOBP1–Pear ester	−1.12 ± 0.01	−35.63 ± 0.01	10.48 ± 0.01	−5.34 ± 0.00	−31.61 ± 0.02
AoraGOBP1–Decanal	−2.62 ± 0.01	−28.95 ± 0.01	8.40 ± 0.01	−4.37 ± 0.00	−27.54 ± 0.02
AoraGOBP1–Methyl salicylate	−1.71 ± 0.01	−21.97 ± 0.01	9.75 ± 0.01	−3.36 ± 0.00	−17.29 ± 0.01
AoraGOBP1–Citral	−0.70 ± 0.02	−26.81 ± 0.01	7.15 ± 0.02	−4.10 ± 0.00	−24.46 ± 0.01
AoraGOBP1–1-Penten-3-ol	−3.00 ± 0.02	−13.32 ± 0.01	6.33 ± 0.02	−3.99 ± 0.00	−12.34 ± 0.02
AoraGOBP1–Dibutyl phthalate	−6.42 ± 0.02	−44.93 ± 0.02	18.69 ± 0.02	−6.04 ± 0.00	−24.46 ± 0.01
AoraGOBP1– Ethyl 2-methylbutanoate	−1.57 ± 0.01	−21.82 ± 0.01	6.75 ± 0.02	−3.34 ± 0.00	−19.97 ± 0.01
AoraGOBP1–Isobutyraldehyde	−1.13 ± 0.01	−12.28 ± 0.01	4.20 ± 0.01	−1.99 ± 0.00	−11.20 ± 0.01
AoraGOBP2–*Z*9-14:Ac	−2.31 ± 0.02	−46.26 ± 0.02	12.07 ± 0.01	−6.69 ± 0.00	−43.18 ± 0.02
AoraGOBP2–*Z*9-14:OH	−4.96 ± 0.02	−38.65 ± 0.02	10.02 ± 0.01	−5.75 ± 0.00	−39.34 ± 0.02
AoraGOBP2–*Z*11-14:OH	−7.86 ± 0.04	−38.41 ± 0.02	12.94 ± 0.03	−5.87 ± 0.00	−39.20 ± 0.02
AoraGOBP2–12:OH	−11.39 ± 0.03	−34.23 ± 0.02	14.49 ± 0.02	−5.39 ± 0.00	−36.53 ± 0.02
AoraGOBP2–Farnesol	−0.82 ± 0.01	−36.78 ± 0.02	7.41 ± 0.01	−5.30 ± 0.00	−35.49 ± 0.02
AoraGOBP2–Farnesene	−3.56 ± 0.02	−39.35 ± 0.02	10.99 ± 0.02	−5.57 ± 0.00	−37.49 ± 0.02
AoraGOBP2–Dibutyl phthalate	−7.13 ± 0.02	−44.73 ± 0.04	19.34 ± 0.01	−6.41 ± 0.01	−38.94 ± 0.02

## Data Availability

Data inquiries can be directed to the corresponding author.

## Supplementary Materials

The following supporting information can be downloaded at: https://www.mdpi.com/article/10.3390/insects16090880/s1, Figure S1: Amino acid sequences of AoraGOBP1 and AoraGOBP2. Signal peptides are signed with underlines; Figure S2: Phylogenetic relationship analysis of GOBPs of *A. arona* and other lepidopteron species; Figure S3: Binding of ligands to AoraGOBPs; Figure S4: Ramachandran plots of AoraGOBP1 and AoraGOBP2 models; Figure S5: 2D plots of molecular docking for the binding of AoraGOBP1 and AoraGOBP2 to plant volatiles and sex pheromones; Figure S6: root-mean-square deviation (RMSD) curves of AoraGOBP–ligand complexes; Figure S7: The root-mean-square fluctuation (RMSF) curves of AoraGOBP–ligand complexes; Table S1: Primers used for gene cloning, RT-qPCR and prokaryotic expression; Table S2: NCBI accession numbers of amino acid sequences for sequence alignment and phylogenetic analyses.

## Abbreviations

The following abbreviations are used in this manuscript:

OBP	Odorant-binding protein
CSP	Chemosensory protein
OR	Odorant receptor
IR	Ionotropic receptor
GR	Gustory receptor
SNMP	Sensory neuron membrane proteins
RT-qPCR	Real-time quantitative polymeric chain reaction
One-way ANOVA	One-way analysis of variance
SDS-PAGE	Sodium dodecyl sulfate polyacrylamide gel electrophoresis
MD simulation	Molecular dynamics simulation
