# Supplementary material for: Functional Divergence of Two General Odorant-Binding Proteins to Sex Pheromones and Host Plant Volatiles in Adoxophyes orana (Lepidoptera: Tortricidae)
Source: Insects. 2025 Aug 24;16(9):880. doi: 10.3390/insects16090880 (PMC12470260; doi:10.3390/insects16090880)
Supplement: Supplementary file 1 [file insects-16-00880-s001.zip › insects-3831101-supplementary.pdf]

>AoraGOBP1
MCGARTLLVLVAVLAAAAATVDVMKDVTLGFGEALQRCRESSQLTEEKMEEFFHFWREDF
KFEHRELGCAIQCMSRHFNLLTDGERMHHDNTDKFIKSFPNGEVLSQQMVALIHSCEQQH
DAEPDHCWRILRVAECFKAGCQARGIAPTMEMLMAEFIMESEA

>AoraGOBP2
MASYWVVCVVLVAGSHLVAGTAEVMSHVTAHFGKALSECREESGLSPAILEEFQHFWREDF
EVVHRELGCAILCMSNKFSLMQDDARMHHENMHDYIKSFPEGEVLSAKMVELIHNCEKQ
YDDIKDDCDRVVKVAACFKVDAKKAGIAPEVAMIEAVMEKY

Figure S1. Amino acid sequences of AoraGOBP1 and AoraGOBP2. Signal peptides are signed with underlines. Cysteines are colored by light blue.

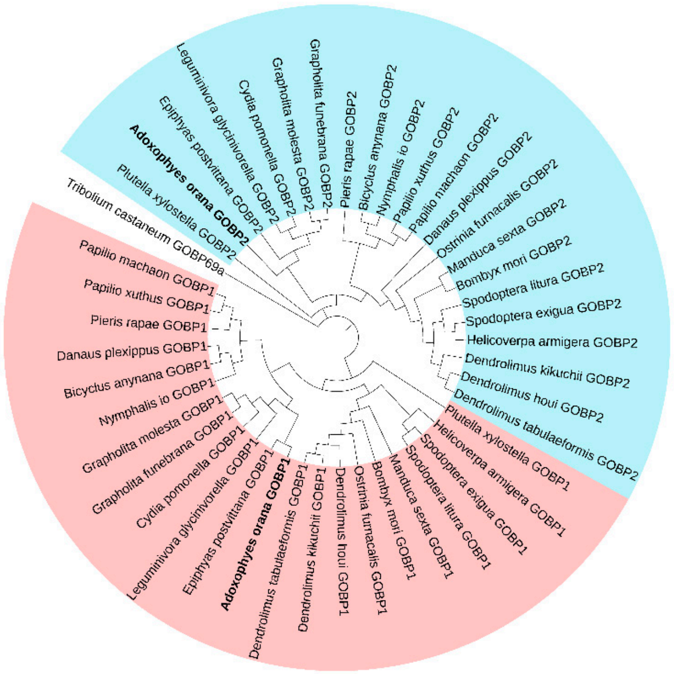

Figure S2. Phylogenetic relationship analysis of GOBPs of *A. arona* and other lepidopteron species. The NCBI accession numbers of amino acids are listed in Table S2.

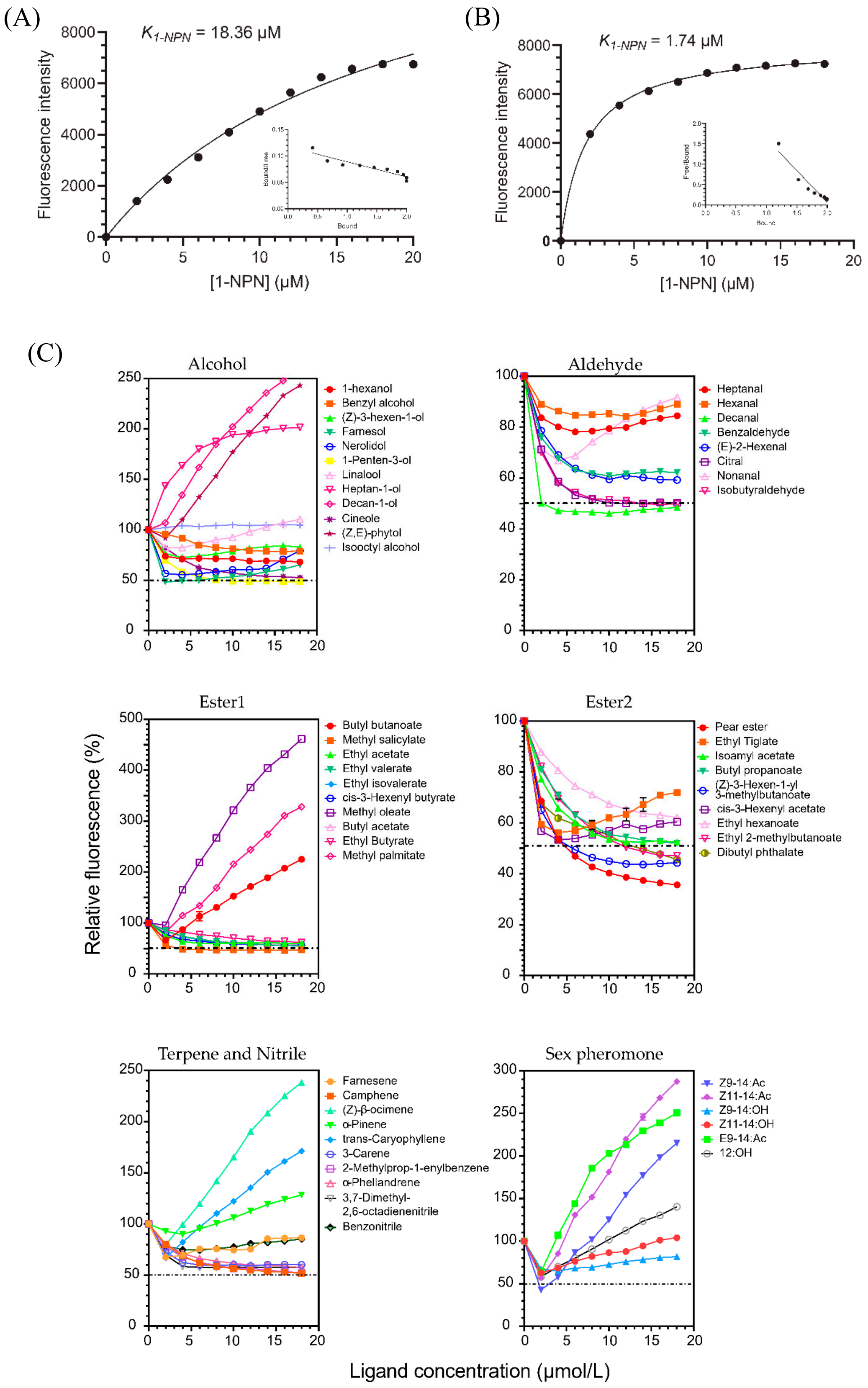

(A)
$K_{1\text{-}NPN}$ = 18.36 μM
Fluorescence intensity
[1-NPN] (μM)
(B)
$K_{1\text{-}NPN}$ = 1.74 μM
Fluorescence intensity
[1-NPN] (μM)
(C)
Relative fluorescence (%)
Ligand concentration (μmol/L)
Alcohol
1-hexanol
Benzyl alcohol
(Z)-3-hexen-1-ol
Farnesol
Nerolidol
1-Penten-3-ol
Linalool
Heptan-1-ol
Decan-1-ol
Cineole
(Z,E)-phytol
Isooctyl alcohol
Aldehyde
Heptanal
Hexanal
Decanal
Benzaldehyde
(E)-2-Hexenal
Citral
Nonanal
Isobutyraldehyde
Ester1
Butyl butanoate
Methyl salicylate
Ethyl acetate
Ethyl valerate
Ethyl isovalerate
cis-3-Hexenyl butyrate
Methyl oleate
Butyl acetate
Ethyl Butyrate
Methyl palmitate
Ester2
Pear ester
Ethyl Tiglate
Isoamyl acetate
Butyl propanoate
(Z)-3-Hexen-1-yl 3-methylbutanoate
cis-3-Hexenyl acetate
Ethyl hexanoate
Ethyl 2-methylbutanoate
Dibutyl phthalate
Terpene and Nitrile
Farnesene
Camphene
(Z)-β-ocimene
α-Pinene
trans-Caryophyllene
3-Carene
2-Methylprop-1-enylbenzene
α-Phellandrene
3,7-Dimethyl-2,6-octadienenitrile
Benzonitrile
Sex pheromone
Z9-14:Ac
Z11-14:Ac
Z9-14:OH
Z11-14:OH
E9-14:Ac
12:OH

(D)

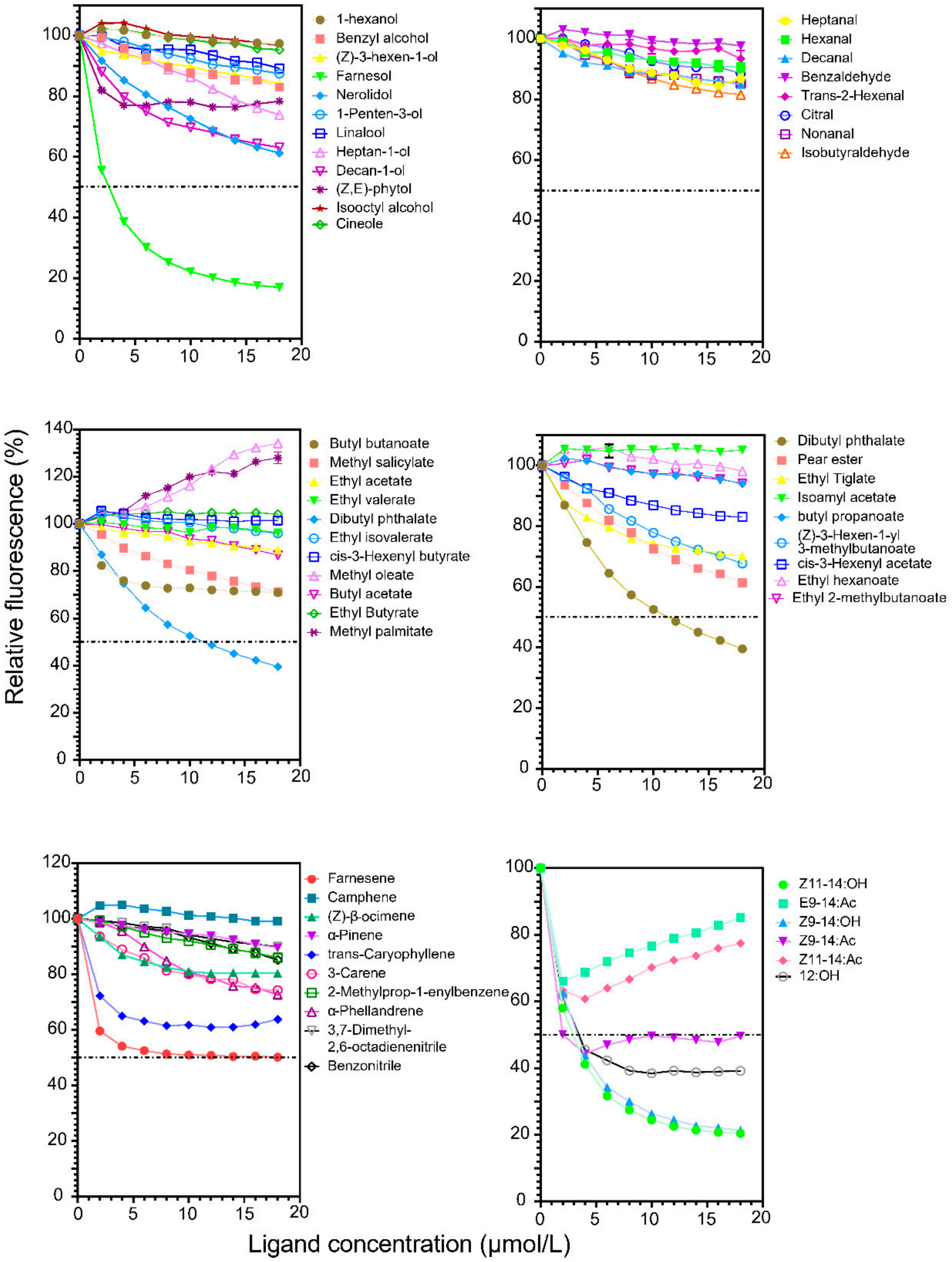

Figure S3. Binding of ligands to AoraGOBPs. **(A)** Binding curve of 1-NPN to AoraGOBP1 with relative Scatchard plots. **(B)** Binding curve of 1-NPN to AoraGOBP2 with relative Scatchard plots. **(C)** Binding curves of host plant volatiles and sex pheromones to AoraGOBP1. **(D)** Binding curves of host plant volatiles and sex pheromones to AoraGOBP2. The binding data of sex pheromones and host plant volatiles were calculated and listed in Table 1.

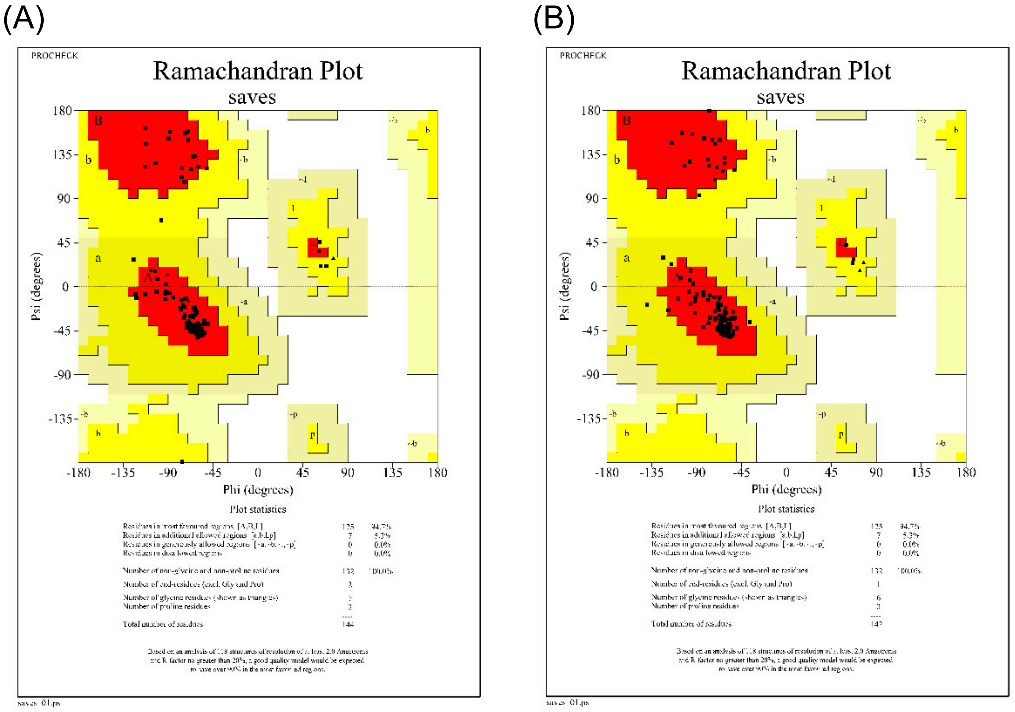

Figure S4. Ramachandran plots of AoraGOBP1 and AoraGOBP2 models. 94.7% **(A)** and 94.7% **(B)** of the residues are in the most favored regions in GOBP1 and GOBP2 models, respectively.

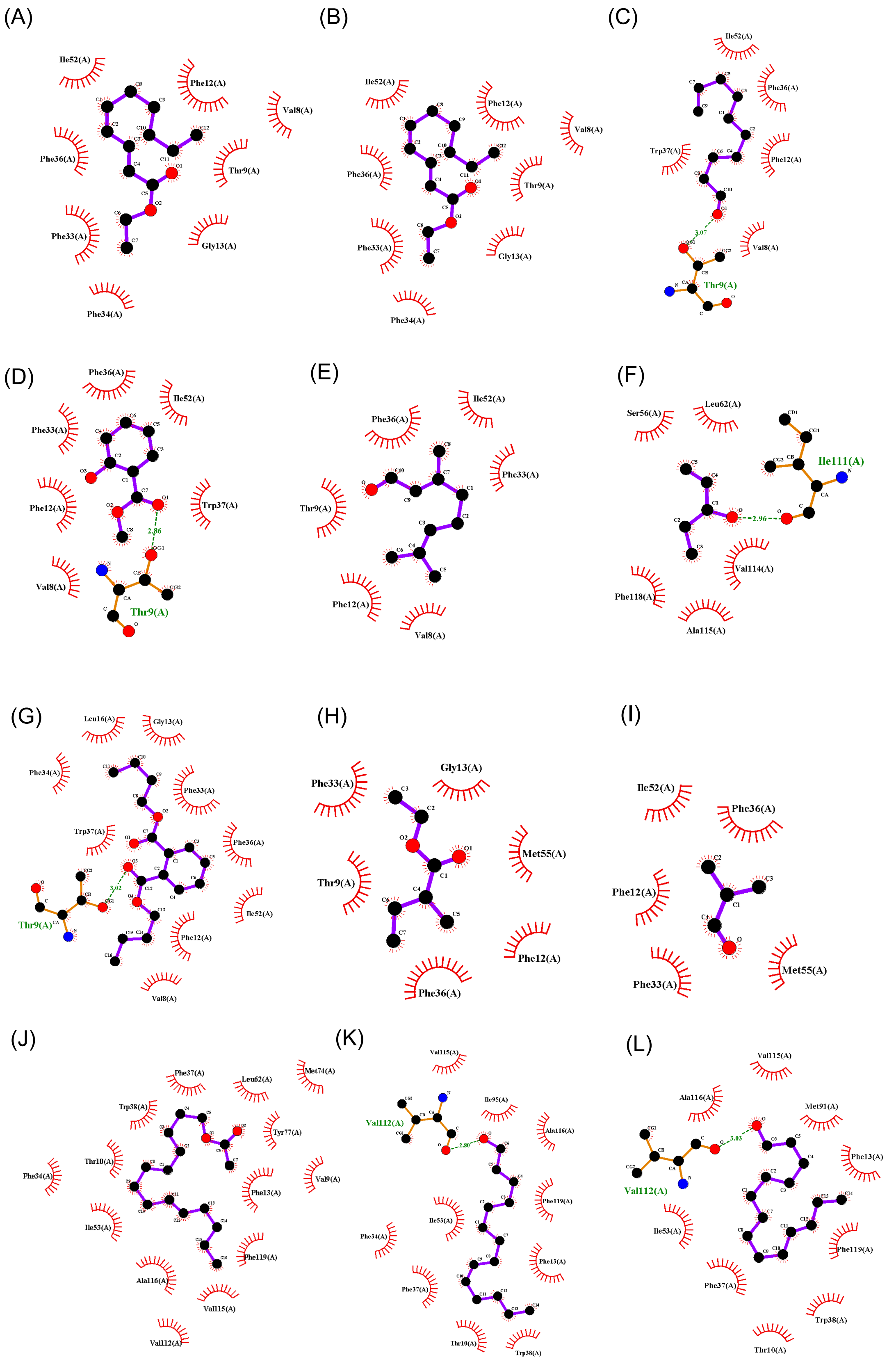

(A)
Ile52(A)
Phe12(A)
Val8(A)
Phe36(A)
Thr9(A)
Phe33(A)
Gly13(A)
Phe34(A)
(B)
Ile52(A)
Phe12(A)
Val8(A)
Phe36(A)
Thr9(A)
Phe33(A)
Gly13(A)
Phe34(A)
(C)
Ile52(A)
Phe36(A)
Trp37(A)
Phe12(A)
3.07
Val8(A)
Thr9(A)
(D)
Phe36(A)
Ile52(A)
Phe33(A)
Phe12(A)
Trp37(A)
2.86
Val8(A)
Thr9(A)
(E)
Ile52(A)
Phe36(A)
Phe33(A)
Thr9(A)
Phe12(A)
Val8(A)
(F)
Ser56(A)
Leu62(A)
Ile111(A)
2.96
Val114(A)
Phe118(A)
Ala115(A)
(G)
Leu16(A)
Gly13(A)
Phe34(A)
Phe33(A)
Trp37(A)
Phe36(A)
3.02
Thr9(A)
Ile52(A)
Phe12(A)
Val8(A)
(H)
Phe33(A)
Gly13(A)
Met55(A)
Thr9(A)
Phe12(A)
Phe36(A)
(I)
Ile52(A)
Phe36(A)
Phe12(A)
Phe33(A)
Met55(A)
(J)
Phe37(A)
Leu62(A)
Met74(A)
Trp38(A)
Tyr77(A)
Thr10(A)
Phe34(A)
Val9(A)
Phe13(A)
Ile53(A)
Phe119(A)
Ala116(A)
Val115(A)
Val112(A)
(K)
Val115(A)
Ile95(A)
Val112(A)
2.80
Ala116(A)
Phe119(A)
Ile53(A)
Phe34(A)
Phe13(A)
Phe37(A)
Thr10(A)
Trp38(A)
(L)
Val115(A)
Ala116(A)
Met91(A)
3.03
Phe13(A)
Val112(A)
Ile53(A)
Phe119(A)
Phe37(A)
Trp38(A)
Thr10(A)

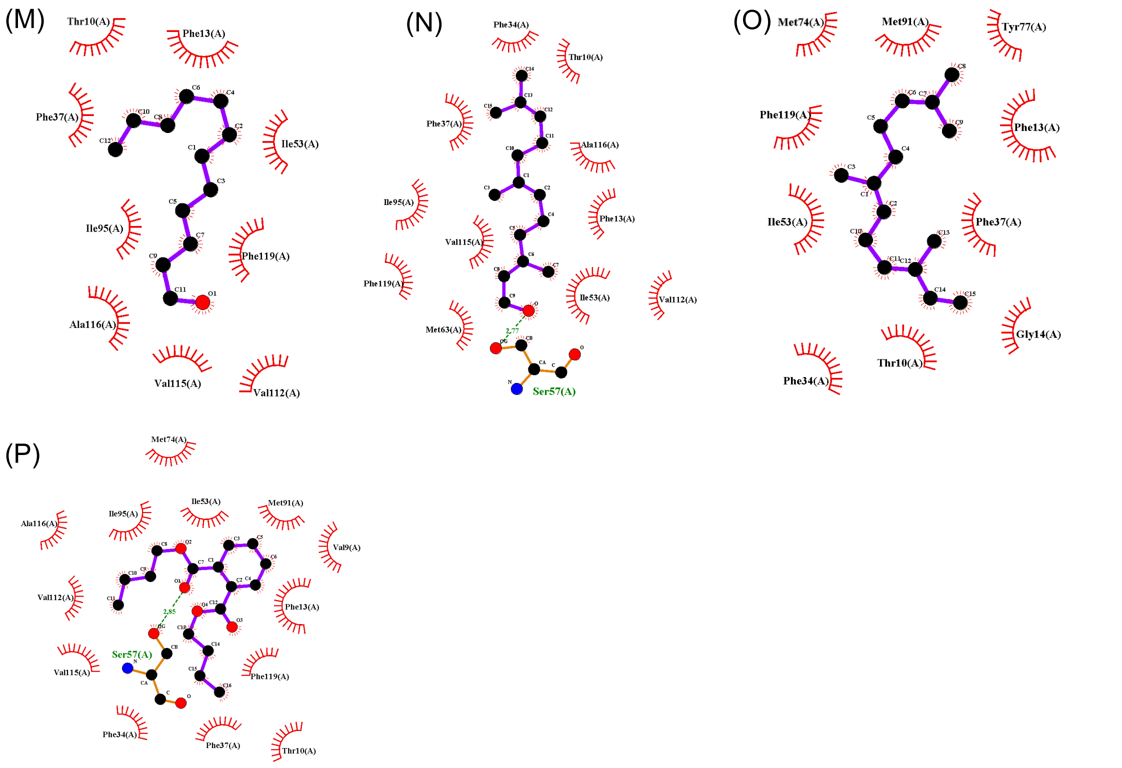

Figure S5. 2D plots of molecular docking for the binding of AoraGOBP1 and AoraGOBP2 to plant volatiles and sex pheromones. **(A)** AoraGOBP1–cis-3-Hexenyl isovalerate. **(B)** AoraGOBP1–Pear ester. **(C)** AoraGOBP1–Decanal. **(D)** AoraGOBP1–Methyl salicylate. **(E)** AoraGOBP1–Citral. **(F)** AoraGOBP1–1-penten-3-ol. **(G)** AoraGOBP1–Dibutyl phthalate. **(H)** AoraGOBP1–Ethyl 2-methylbutyrate. **(I)** AoraGOBP1–Isobutyraldehyde. **(J)** AoraGOBP2–Z9-14:Ac. **(K)** AoraGOBP2–Z9-14:OH. **(L)** AoraGOBP2–Z11-14:OH. **(M)** AoraGOBP2–12:OH. **(N)** AoraGOBP2–Farnesol. **(O)** AoraGOBP2–Farnesene. **(P)** AoraGOBP2–Dibutyl Phthalate. The green, magentas, and purple dashed lines represent hydrogen bonds, π-π stacks, and π-σ interactions, respectively

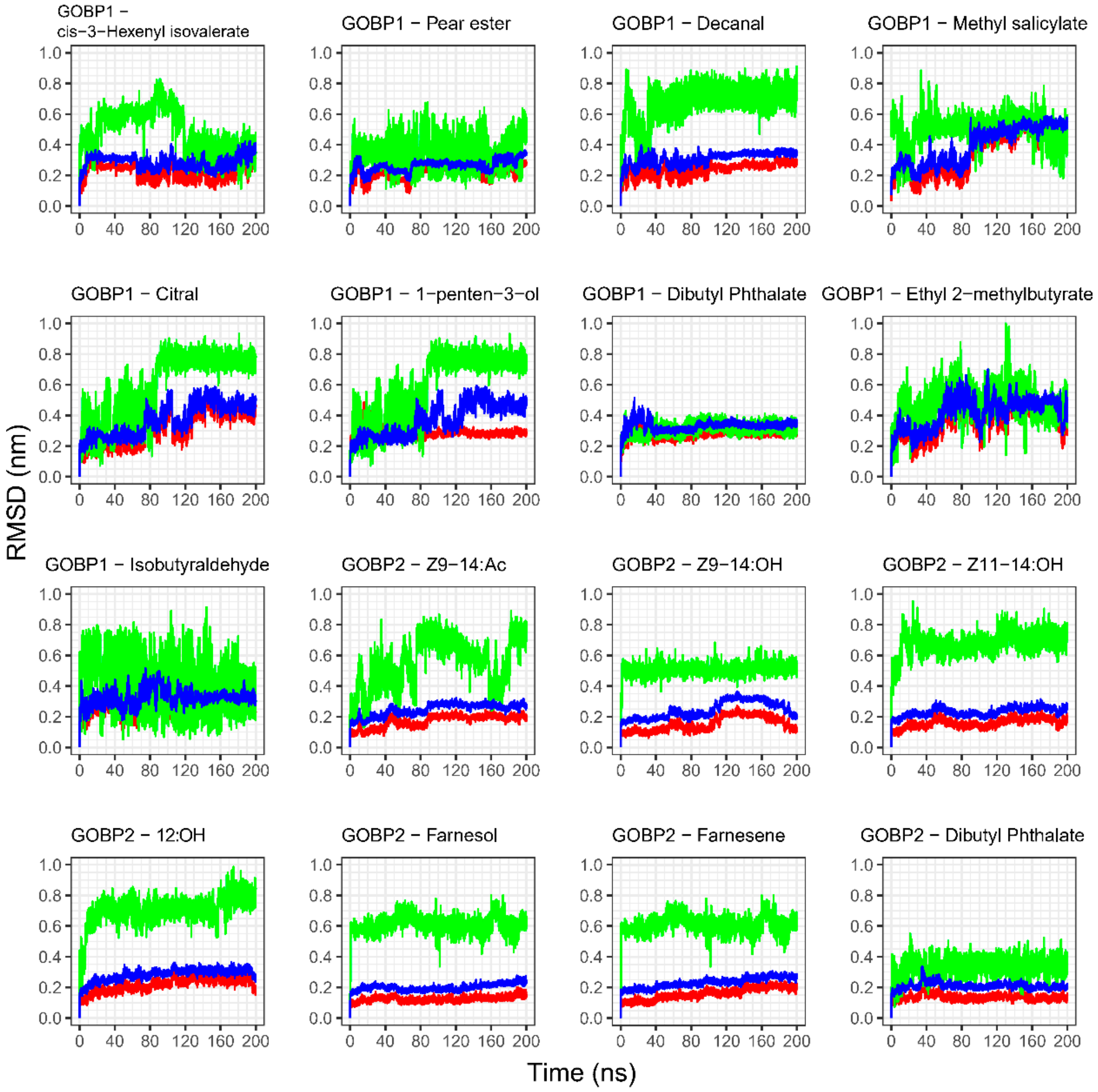

Figure S6. The root-mean-square deviation (RMSD) curves of AoraGOBP-ligand complexes. Red, green, and blue curves represent RMSD of protein, ligand, and complex, respectively.

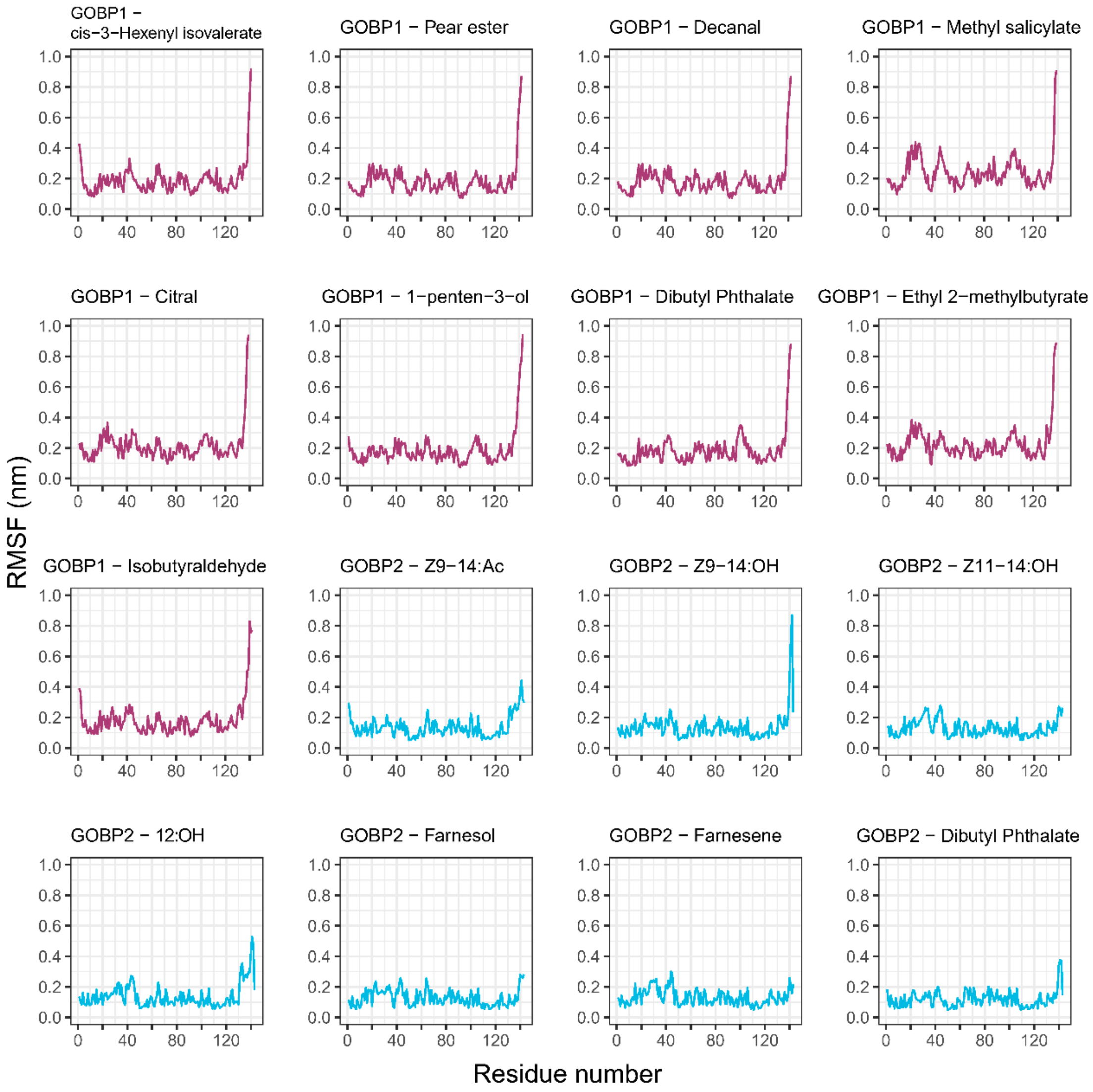

Figure S7. The root-mean-square fluctuation (RMSF) curves of AoraGOBP-ligand complexes.

Table S1. Primers used for gene cloning, RT-qPCR and prokaryotic expression.

Gene	Reverse	Forward primier
AoraGOBP1	forward strand	GTAACTCCGGCGACTCCTAC
	reverse strand	TGGTAGCCCTAAGAGCGAGT
qAoraGOBP1	forward strand	TTCAAGTTCGAGCACAGCGA
Reverse strand	reverse strand	GCCGTTAGGGAAGGACTTGA
eAoraGOBP1	forward strand	CGGGATCCACCGTCGACGTCATGAAGGATG (BamHI)
	reverse strand	CCCAAGCTTGGGTTAAGCCTCAGACTCCATGATG (HindIII)
AoraGOBP2	forward strand	TCATGGCTTCTTACTGGGTG
	reverse strand	GAGGTGACCCTTCGTAAACA
qAoraGOBP2	forward strand	GCTTCCCTGAAGGTGAGGTC
	reverse strand	GCGATGCCTGCTTTCTTAGC
eAoraGOBP2	forward strand	CGGGATCCACAGCAGAGGTCATGAGCCATG (BamHI)
	reverse strand	CCCAAGCTTGGGTTAATATTTCTCCATGACAGC (HindIII)

Table S2. NCBI accession numbers of amino acid sequences for sequence alignment and phylogenetic analyses

GOBP name	Species	Accession number	GOBP name	Species	Accession number
CpomGOBP1	*Cydia pomonella*	XP_061721229.1	CpomGOBP2	*Cydia pomonella*	XP_061721229.1
GfunGOBP1	*Grapholita funebrana*	URZ86309.1	GfunGOBP1	*Grapholita funebrana*	URZ86310.1
LglyGOBP1	*Leguminivora glycinivorella*	AMP46760.1	LglyGOBP1	*Leguminivora glycinivorella*	XP_047997209.1
GmolGOBP1	*Grapholita molesta*	AFH02841.1	GmolGOBP2	*Grapholita molesta*	AFH02842.1
SlitGOBP1	*Spodoptera litura*	XP_022816701.1	SlitGOBP2	*Spodoptera litura*	AKI87961.1
SexiGOBP1	*Spodoptera exigua*	ACY78412.1	SexiGOBP2	*Spodoptera exigua*	AGH70098.1
HarmGOBP1	*Helicoverpa armigera*	XP_021192665.3	HarmGOBP2	*Helicoverpa armigera*	XP_063894329.1
PxylGOBP1	*Plutella xylostella*	ABY71034.1	PxylGOBP2	*Plutella xylostella*	ABY71035.2
OfurGOBP1	*Ostrinia furnacalis*	BAV56786.1	OfurGOBP2	*Ostrinia furnacalis*	ABG66419.2
BmorGOBP1	*Bombyx mori*	CAA64444.1	BmorGOBP2	*Bombyx mori*	CAA64445.1
DtabGOBP1	*Dendrolimus tabulaeformis*	AGJ71276.1	DtabGOBP2	*Dendrolimus tabulaeformis*	AGJ71277.1
DhouGOBP1	*Dendrolimus houi*	AGJ83358.1	DhouGOBP1	*Dendrolimus houi*	AGJ83354.1
DkikGOBP1	*Dendrolimus kikuchii*	AGJ83357.1	DkikGOBP2	*Dendrolimus kikuchii*	AGJ83353.1
PmacGOBP1	*Papilio machaon*	XP_014368564.2	PmacGOBP2	*Papilio machaon*	XP_014368562.2
PxutGOBP1	*Papilio xuthus*	KPJ00898.1	PxutGOBP2	*Papilio xuthus*	XP_013162827.1
EposGOBP1	*Epiphyas postvittana*	JAI18134.1	EposGOBP2	*Epiphyas postvittana*	AAL05869.1
MsexGOBP1	*Manduca sexta*	AAA29315.1	MsexGOBP2	*Manduca sexta*	KAG6450958.1
DpleGOBP1	*Danaus plexippus*	XP_032519594.2	DplexGOBP2	*Danaus plexippus*	XP_032519593.2
ObruGOBP1	*Operophtera brumata*	KOB70852.1			
